# Recyclable Magnetic Iron Immobilized onto Chitosan with Bridging Cu Ion for the Enhanced Adsorption of Methyl Orange

**DOI:** 10.3390/molecules28052307

**Published:** 2023-03-02

**Authors:** Daoguang Teng, Peng Jin, Wenhuan Guo, Jiang Liu, Wei Wang, Peng Li, Yijun Cao, Ling Zhang, Ying Zhang

**Affiliations:** 1 Zhongyuan Critical Metals Laboratory, Zhengzhou University, Zhengzhou 450001, China; 2 School of Chemical Engineering, Zhengzhou University, Zhengzhou 450001, China

**Keywords:** magnetic, chitosan, bridging Cu, recyclable adsorbent, methyl orange

## Abstract

Chitosan (CS) is a natural and low-cost adsorbent for capturing metal ions and organic compounds. However, the high solubility of CS in acidic solution would make it difficult to recycle the adsorbent from the liquid phase. In this study, the CS/Fe_3_O_4_ was prepared via Fe_3_O_4_ nanoparticles immobilized onto a CS surface, and the DCS/Fe_3_O_4_-Cu was further fabricated after surface modification and the adsorption of Cu ions. The meticulously tailored material displayed the sub-micron size of an agglomerated structure with numerous magnetic Fe_3_O_4_ nanoparticles. During the adsorption of methyl orange (MO), the DCS/Fe_3_O_4_-Cu delivered a superior removal efficiency of 96.4% at 40 min, which is more than twice the removal efficiency of 38.7% for pristine CS/Fe_3_O_4_. At an initial MO concentration of 100 mg L^−1^, the DCS/Fe_3_O_4_-Cu exhibited the maximum adsorption capacity of 144.60 mg g^−1^. The experimental data were well explained by the pseudo-second-order model and Langmuir isotherm, suggesting the dominant monolayer adsorption. The composite adsorbent still maintained a large removal rate of 93.5% after five regeneration cycles. This work develops an effective strategy to simultaneously achieve high adsorption performance and convenient recyclability for wastewater treatment.

## 1. Introduction

Increasingly discharged industrial wastewater from textile mills and metal electroplating plants greatly endanger the whole ecosystem with the rapid development of industrialization [1]. Contaminants, especially heavy metal ions (e.g., Cu^2+^) and organic dyes (e.g., methylene orange, MO), seriously threaten living organisms even at trace levels through binding with enzymes and proteins [2,3]. Therefore, efficient wastewater treatment concerning Cu^2+^ and MO removal is urgently needed. At present, various methods were used to remove metal ions and organic compounds from solution, including chemical precipitation, ion exchange, membrane separation, electrochemical method, redox method and adsorption [4,5]. Among these approaches, due to simple operation, affordable price and high efficiency, adsorption was widely applied to uptake contaminants in wastewater [6].

Recently, natural organic or inorganic adsorbents have attracted increasingly attention owing to the plentiful sources, low cost and environmental friendliness compared with other artificial adsorbents, such as activated carbon, bentonite, and biochar [7]. Chitosan (CS) is a renewable polysaccharide from the deacetylation of natural chitin (in the skeleton of arthropods and cell wall of fungi), which the resource reserve ranks second in nature (only less than cellulose) [8]. The CS shows good biodegradation and functional activity, and it can be regarded as a promising adsorbent material [9]. However, the high solubility of CS in acidic solution would make it is difficult to recycle the CS-based adsorbent from the liquid phase. Using surface modification to introduce magnetic material (e.g., Fe_3_O_4_) into CS would address the recovery issue [10]. Some researchers reported the removal of heavy metal ions (such as Cu^2+^, Co^2+^, Ni^2+^, and Pb^2+^) by the magnetic CS material with considerable adsorption capacity [11,12]. Moreover, the modified magnetic CS was also applied as adsorbent to remove organic dyes (such as blue 21, food yellow 3, and acid yellow 23) from wastewater [13]. The above reports usually used the fresh CS-based adsorbent, while there was almost no report using the spent adsorbent after the adsorption of contaminants. It is still a huge challenge to adsorb organic dye using the spent CS-based adsorbent after the removal of heavy metal ion.

**Figure 1:**
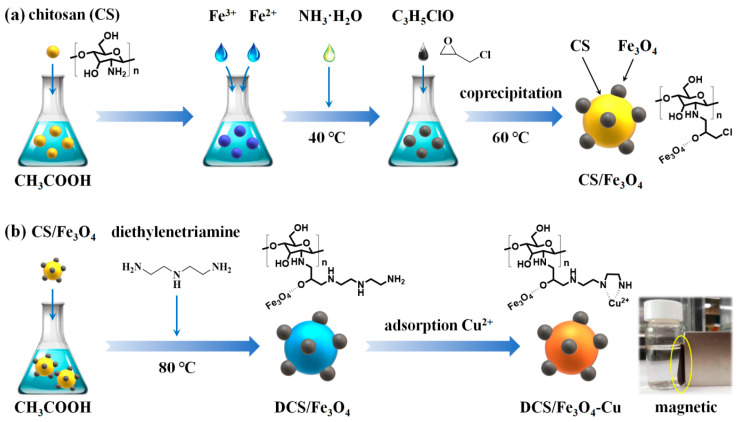
Schematic diagram of synthesis process for (**a**) CS/Fe_3_O_4_, (**b**) DCS/Fe_3_O_4_-Cu.

In this work, the Fe_3_O_4_ nanoparticles immobilized onto CS (CS/Fe_3_O_4_) were prepared via the facile coprecipitation method (Figure 1a). Using diethylenetriamine for the surface modification of CS to provide abundant amino groups (–NH_2_), the CS transformed to DCS. During the adsorption of Cu ions, these amino groups could bridge Cu^2+^, and the DCS/Fe_3_O_4_ further formed the spent adsorbent (defined as DCS/Fe_3_O_4_-Cu). Such a meticulously tailored adsorbent exhibits several advantages [14,15]: (a) the adsorbent can maintain the stable particle structure in solution; (b) the magnetic adsorbent can be easily separated and recycled from the liquid phase environment; and (c) the bridging cationic Cu^2+^ can adsorb anionic organic matter (e.g., MO anions). Accordingly, the DCS/Fe_3_O_4_-Cu material delivered enhanced adsorption capability of MO compared with the pristine CS/Fe_3_O_4_ adsorbent. Furthermore, the kinetics, isotherm and thermodynamics were also investigated to reveal the adsorption mechanism. The purpose of the present study was to devise an available method for preparing a recyclable CS-based adsorbent with low cost and high adsorption performance.

## 2. Results and Discussion

### 2.1. Physical Properties of DCS/Fe_3_O_4_-Cu

**Figure 2:**
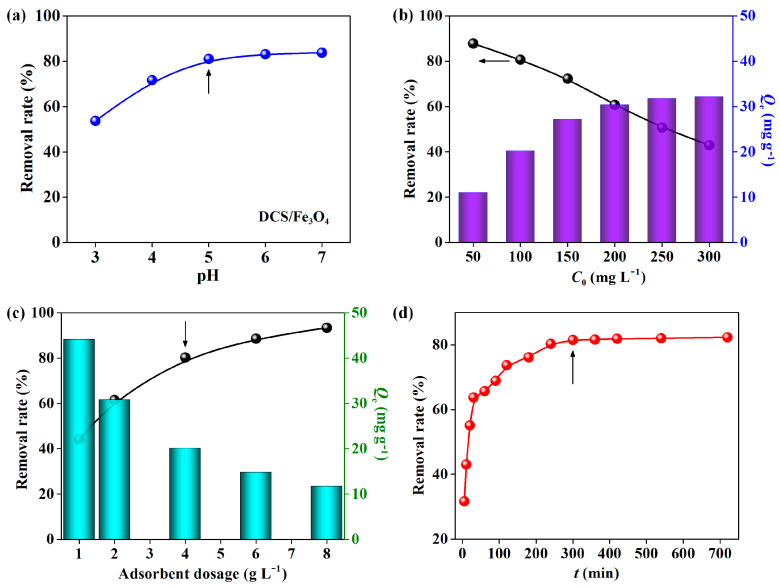
Effect of (**a**) pH value, (**b**) initial Cu^2+^ concentration, (**c**) absorbent dosage, and (**d**) contact time on Cu^2+^ removal efficiency for DCS/Fe_3_O_4_.

Figure 2 presents the effects of adsorption conditions on Cu^2+^ removal efficiency for the DCS/Fe_3_O_4_ material [16]. From Figure 2a, the Cu^2+^ removal efficiency increased with the reduction in pH value. At pH = 5, the removal efficiency reached the saturated value (>80%). Figure 2b showed that the Cu^2+^ removal rate decreased with the increasement of initial Cu^2+^ concentrations (50–300 mg L^−1^), while the corresponding adsorption capacity increased. As seen in Figure 2c, with the absorbent dosage increased from 1 to 8 g L^−1^, the Cu^2+^ removal rate increased from 44.1% to 93.4%. With the prolonging of contact time, the Cu^2+^ removal efficiency gradually increased (Figure 2d). Under the optimal conditions of pH = 5, initial Cu^2+^ concentration = 100 mg L^−1^, absorbent dosage = 4 g L^−1^, and contact time = 300 min, the removal efficiency of Cu^2+^ could reach >80%. The spent adsorbent DCS/Fe_3_O_4_-Cu was collected and further used for the subsequent adsorption of MO. Similarly, the effects of adsorption conditions on Cu^2+^ removal efficiency for the CS/Fe_3_O_4_ were also conducted. As seen from Figure S1 (Supplementary Materials), the optimal conditions for CS/Fe_3_O_4_ to remove Cu^2+^ were pH = 5, initial Cu^2+^ concentration = 100 mg L^−1^, absorbent dosage = 4 g L^−1^, and contact time = 300 min, while the removal efficiency was only recorded as ~60%.

**Figure 3:**
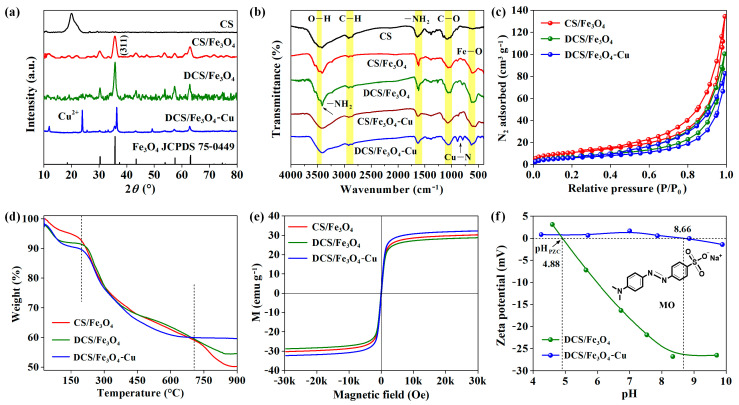
(**a**) XRD patterns of CS, CS/Fe_3_O_4_, DCS/Fe_3_O_4_, and DCS/Fe_3_O_4_-Cu, (**b**) FTIR spectra of five samples, (**c**) N_2_ adsorption–desorption isotherms of CS/Fe_3_O_4_, DCS/Fe_3_O_4_, and DCS/Fe_3_O_4_-Cu, (**d**) TG curves of three samples, (**e**) magnetic hysteresis loops of three samples, (**f**) zeta potential of DCS/Fe_3_O_4_ and DCS/Fe_3_O_4_-Cu.

The X-ray diffraction (XRD) patterns of CS, CS/Fe_3_O_4_, DCS/Fe_3_O_4_, and DCS/Fe_3_O_4_-Cu are illustrated in Figure 3a. The CS sample showed a wide peak located at 2*θ* = ~21.5°, which was a typical characteristic of semi-crystalline CS [9]. For the CS/Fe_3_O_4_ sample, several new peaks appeared at around 30.3°, 36.2°, 43.2°, 57.4°, and 63.1°, corresponding to the (220), (311), (400), (511), and (440) planes of cubic Fe_3_O_4_ lattice (JCPDS. 75-0449) [17]. The characteristic peak of CS disappeared in the CS/Fe_3_O_4_ pattern due to the disordered crystal structures that resulted from interactions of Fe_3_O_4_ nanoparticles with the CS matrix [17]. For the DCS/Fe_3_O_4_ and DCS/Fe_3_O_4_-Cu, both samples maintained the diffraction peaks of Fe_3_O_4_, suggesting the structural integrity of composite materials. On the DCS/Fe_3_O_4_-Cu pattern, a new sharp peak at ~24.0° could be attributed to the bridging Cu^2+^ [18]. Using the Scherrer equation and Segal method, the grain size and crystallinity index could be calculated in Table S1. The wide diffraction peak of CS suggested its low crystallinity (grain size of 3.4 nm and crystallinity index of 63%). The DCS/Fe_3_O_4_-Cu sample exhibited high crystallinity (grain size of 27.9 nm and crystallinity index of 86%).

The Fourier-transform infrared (FTIR) spectra of CS, CS/Fe_3_O_4_, DCS/Fe_3_O_4_, CS/Fe_3_O_4_-Cu and DCS/Fe_3_O_4_-Cu samples are shown in Figure 3b. Two bands located at around 3480 and 2900 cm^−1^ were assigned to the stretching vibration of hydroxyl groups (O–H) and C–H bond, respectively [9]. The bands near 1640 and 1080 cm^−1^ could be related to the bending vibration of amino groups (–NH_2_) and stretching vibration of the C–O bond in CS molecules [17]. Apart from the pure CS sample, the new band that appeared at approximately 600 cm^−1^ in other four samples corresponded to the stretching vibration of the Fe–O bond [19]. For the DCS/Fe_3_O_4_ sample, a sharp peak at ~3420 cm^−1^ was assigned to the stretching vibration of –NH_2_ from diethylenetriamine molecule. After Cu^2+^ adsorption, the peak at 3420 cm^−1^ disappeared, and the DCS/Fe_3_O_4_-Cu sample expressed a new band at about 890 cm^−1^, attributing to the interaction between bridging Cu ions and –NH_2_ groups (Cu–N) [20,21]. While for the CS/Fe_3_O_4_ sample, there was no similar Cu–N bond on the spectrum.

Liquid nitrogen adsorption–desorption isotherms of CS/Fe_3_O_4_, DCS/Fe_3_O_4_ and DCS/Fe_3_O_4_-Cu are shown in Figure 3c. All three samples presented the type IV isotherms with an obvious hysteresis loop. The hysteresis loop indicated the existence of abundant mesoporous structures (pore diameter from 2 to 50 nm) in materials [22]. According to the classical Brunauer–Emmett–Teller (BET) equation, the specific surface areas of CS/Fe_3_O_4_, DCS/Fe_3_O_4_ and DCS/Fe_3_O_4_-Cu samples were determined as 38.4, 28.7, and 25.8 m^2^ g^−1^, respectively. The pore texture parameters of above three samples can be found in Table S2. Moreover, Figure S2 shows the Barrett–Joyner–Halenda (BJH) pore size distributions of these samples [9]. The CS/Fe_3_O_4_ material presented the largest pore volume of 0.198 cm^3^ g^−1^ with an average pore diameter of 20.6 nm, while the DCS/Fe_3_O_4_-Cu sample presented the smallest pore volume of 0.142 cm^3^ g^−1^ (average pore diameter of 22.0 nm). The small specific surface area and pore volume of DCS/Fe_3_O_4_-Cu indicated that modified molecules blocked or occupied part of the pores of the CS/Fe_3_O_4_ material [22].

The thermal behaviors of the CS/Fe_3_O_4_, DCS/Fe_3_O_4_, and DCS/Fe_3_O_4_-Cu samples were studied by thermogravimetric (TG) analysis (Figure 3d). The results showed a small decline below 180 °C in the TG curves, corresponding to the evaporation of adsorbed water molecules. A significant decline ranging from 180 to 700 °C could be ascribed to the pyrolysis of chitosan [23]. Subsequently, the TG curves were basically stable above 700 °C. Among three samples, the CS/Fe_3_O_4_ and DCS/Fe_3_O_4_ expressed the large weight loss, suggesting their high carbon contents. For the DCS/Fe_3_O_4_-Cu sample, its small weight loss could be attributed to bridging Cu ions.

The magnetic hysteresis loops of CS/Fe_3_O_4_, DCS/Fe_3_O_4_ and DCS/Fe_3_O_4_-Cu are displayed in Figure 3e. The magnetic properties of adsorbents confirmed that Fe^3+^ and Fe^2+^ can form magnetic Fe_3_O_4_ particles in an alkaline environment. The saturation magnetizations of as-prepared samples were all beyond 27 emu g^−1^, which were convenient for the separation and recycling of spent adsorbents from the liquid phase environment [17].

The zeta potential curves (Figure 3f) can give the pH_PZC_ values (pH value at zeta potential of 0) of DCS/Fe_3_O_4_ and DCS/Fe_3_O_4_-Cu. The sample surface exhibited negative charges when the pH > pH_PZC_; and it exhibited positive charges when the pH < pH_PZC_ [24]. The pH_PZC_ value of the DCS/Fe_3_O_4_ material was fitted as 4.88. Due to the bridging Cu^2+^, the pH_PZC_ value of the DCS/Fe_3_O_4_-Cu sample shifted to 8.66. During the MO adsorption process, the MO solution exhibited weak basicity. In this case, the DCS/Fe_3_O_4_ adsorbent would have negative charges to repel MO anions. The positively charged DCS/Fe_3_O_4_-Cu adsorbent would incline to adsorb MO anions, and the small zeta potential (nearly zero) made DCS/Fe_3_O_4_-Cu particles agglomerate in solution (digital photo in Figure 1) [25].

**Figure 4:**
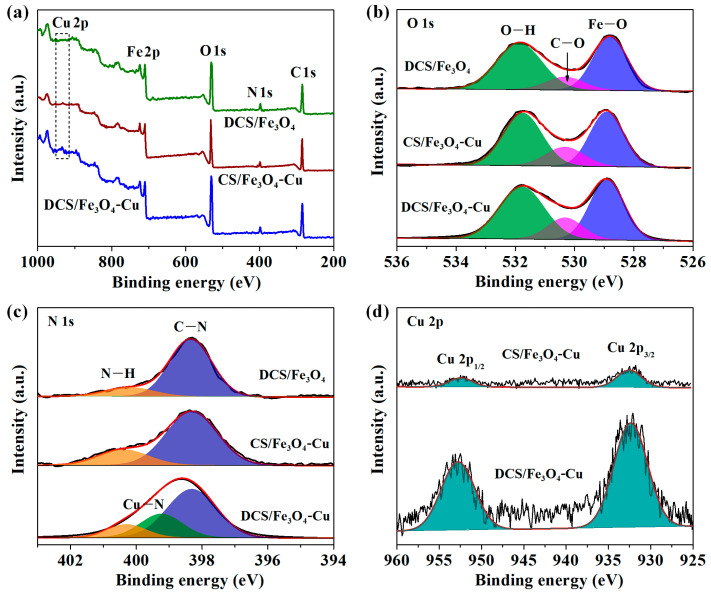
(**a**) XPS survey spectra of DCS/Fe_3_O_4_, CS/Fe_3_O_4_-Cu and DCS/Fe_3_O_4_-Cu, high-resolution XPS spectra of (**b**) O 1s, (**c**) N 1s, (**d**) Cu 2p.

The X-ray photoelectron spectroscopy (XPS) survey spectra of DCS/Fe_3_O_4_, CS/Fe_3_O_4_-Cu and DCS/Fe_3_O_4_-Cu samples are present in Figure 4a. Characteristic peaks at around 285, 399, 531 and 710 eV were attributed to the C 1s, N 1s, O 1s and Fe 2p, respectively [26]. A new peak that appeared at ~934 eV in the DCS/Fe_3_O_4_-Cu spectrum was assigned to Cu 2p.

The O 1s high-resolution XPS spectra in Figure 4b demonstrated three components: peaks at around 531.9, 530.3 and 528.9 eV could be assigned to the Fe–O, C–O and O–H, respectively [27]. The N 1s high-resolution XPS spectra of the DCS/Fe_3_O_4_ and CS/Fe_3_O_4_-Cu samples in Figure 4c could be deconvoluted into two peaks at 398.3 and 400.3 eV, which were possibly related to the pyrrole N of C–N and N–H, respectively [20,27]. A new peak appeared at 399.2 eV in the DCS/Fe_3_O_4_-Cu curve, which resulted from the coordination between Cu ions and amino groups (Cu–N) [20,28]. From Figure 4d, the Cu 2p high-resolution XPS spectrum of DCS/Fe_3_O_4_-Cu sample displayed two obvious spin-orbit doublet peaks of Cu 2p_3/2_ (932.4 eV) and Cu 2p_1/2_ (953.1 eV), corresponding to the bridging Cu ions [20,29]. While for the CS/Fe_3_O_4_-Cu sample, the weak Cu 2p peaks could be assigned to the trace Cu ions adsorbed on the adsorbent surface.

**Figure 5:**
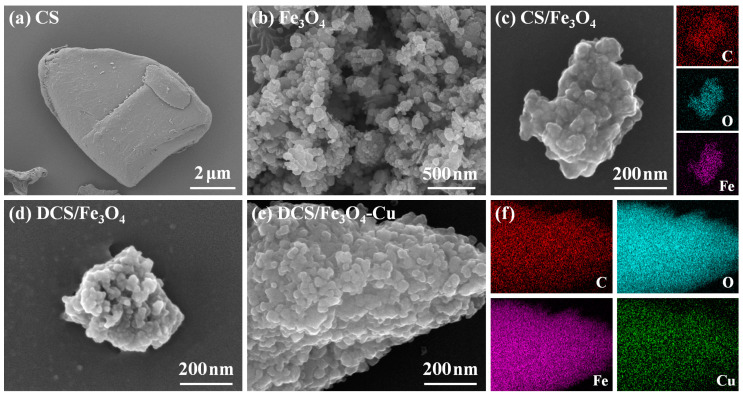
SEM images of (**a**) CS, (**b**) Fe_3_O_4_, (**c**) CS/Fe_3_O_4_ with corresponding mapping images, (**d**) DCS/Fe_3_O_4_, (**e**) DCS/Fe_3_O_4_-Cu, (**f**) mapping images of DCS/Fe_3_O_4_-Cu.

Figure 5 shows the scanning electron microscopy (SEM) images of CS, Fe_3_O_4_, CS/Fe_3_O_4_, DCS/Fe_3_O_4_ and DCS/Fe_3_O_4_-Cu samples. As shown in Figure 5a, the CS sample displayed the irregular particle morphology. The nano-Fe_3_O_4_ material presented an irregular nanosphere shape with the particle size of 50–100 nm (Figure 5b). For the CS/Fe_3_O_4_ sample, Figure 5c exhibits an agglomerated structure, in which numerous Fe_3_O_4_ particles are immobilized onto the surface of the chitosan matrix. The corresponding mapping images and energy-dispersive spectrometer (EDS) spectrum (Figure S3) of the CS/Fe_3_O_4_ sample confirmed the existence of the Fe element [12]. After diethylenetriamine modification and Cu^2+^ adsorption, the DCS/Fe_3_O_4_ (Figure 5d) and DCS/Fe_3_O_4_-Cu (Figure 5e) also maintained the original agglomerated structure. The mapping images (Figure 5f) and EDS spectrum (Figure S3) of the DCS/Fe_3_O_4_-Cu sample further verified the presence of the Cu element [30]. The sub-micron size of DCS/Fe_3_O_4_-Cu can make the adsorbent powder easily separated and recycled from solution [9].

### 2.2. Adsorption of MO on DCS/Fe_3_O_4_-Cu

The effect of light scattering from adsorbent particles was investigated by the UV-Vis absorbance spectra of MO. From Figure S4, with the prolonging of time, the intensity of the MO peak gradually decreased, and the peak shifted a little. All concentration data were calculated according to the peak position.

**Figure 6:**
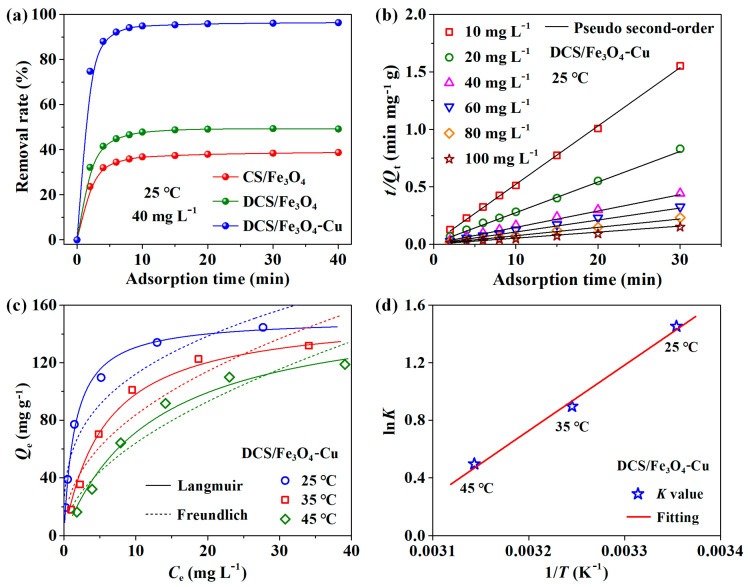
Adsorption data of MO: (**a**) comparison of removal rate for CS/Fe_3_O_4_, DCS/Fe_3_O_4_, and DCS/Fe_3_O_4_-Cu, (**b**) linear fitting of pseudo-second-order kinetic model for DCS/Fe_3_O_4_-Cu, (**c**) adsorption isotherms of DCS/Fe_3_O_4_-Cu at different temperatures, (**d**) thermodynamics plots of ln*K* versus 1/*T* of DCS/Fe_3_O_4_-Cu.

The comparison of MO removal efficiency (initial MO concentration of 40 mg L^−1^, 25 °C) for CS/Fe_3_O_4_, DCS/Fe_3_O_4_ and DCS/Fe_3_O_4_-Cu adsorbents was exhibited in Figure 6a. For all curves, the removal rate increased rapidly in the first 10 min and then gradually reached the adsorption–desorption equilibrium status at an adsorption time of 40 min. Among three adsorbents, the pristine CS/Fe_3_O_4_ sample had the minimum removal efficiency (38.7%). Due to the bridging Cu cations, the DCS/Fe_3_O_4_-Cu material delivered the maximum removal rate of 96.4% for MO anions (more than twice that of CS/Fe_3_O_4_) [31].

Adsorption kinetics was used to investigate the effect of MO concentration. The classic pseudo first-order (Equation (1)) and pseudo second-order (Equation (2)) equations were given as [32]:

(1) $$\ln\left(Q_{e1}-Q_{t}\right)=\ln Q_{e1}-K_{1}\cdot t$$

(2) $$\frac{t}{Q_{t}}=\frac{1}{K_{2}\cdot Q_{e2}^{2}}+\frac{t}{Q_{e2}}$$

where *Q*_t_ (mg g^−1^ min^−1^) is the amount of MO adsorbed at time *t* (min), *K*_1_ (min^−1^) and *K*_2_ (g mg^−1^·min^−1^) are the rate constants of pseudo first-order and pseudo second-order equations.

Using the linear fitting method, the kinetic parameters of MO adsorption onto the DCS/Fe_3_O_4_-Cu sample at 25 °C are listed in Table S3. With MO initial concentration increased from 10 to 100 mg L^−1^, the pseudo-second-order plots all expressed the good fitting degree (Figure 6b), with a high correlation coefficient (*R*^2^) of ~0.998 [33]. Meanwhile, as seen in Figure S5, the fitting degree of the pseudo-first-order model was relatively low. The good fitting of the pseudo-second-order model indicated that the adsorption rate of MO molecules onto the DCS/Fe_3_O_4_-Cu adsorbent was dependent on the amount of adsorption sites rather than the MO concentration in solution [34].

Langmuir and Freundlich isotherms were usually applied to evaluate the adsorption process. The Langmuir model was based on an assumption of homogenous monolayer adsorption, while the Freundlich model was attributed to the heterogeneous multilayer adsorption [35]. The equations of Langmuir (Equation (3)) and Freundlich (Equation (4)) can be expressed as the following [36]:

(3) $$\frac{C_{e}}{Q_{e}}=\frac{1}{Q_{m}\cdot K_{L}}+\frac{C_{e}}{Q_{m}}$$

(4) $$\ln Q_{e}=\ln K_{F}+\frac{\ln C_{e}}{n}$$

where *Q*_m_ (mg g^−1^) is the maximum adsorption capacity at the monolayer, and *K*_L_ (L mg^−1^) is a constant related to the free energy of Langmuir adsorption; *K*_F_ (mg g^−1^) (mg L^−1^)^n^ is the Freundlich constant, and *n* (dimensionless) is the heterogeneity factor.

Figure S6 showed the linear fitting data of MO adsorption onto the DCS/Fe_3_O_4_-Cu adsorbent at 25, 35 and 45 °C. From Figure S6a, the Langmuir model gave a high fitting degree (*R*^2^ > 0.97 in Table S4), while the *R*^2^ values of the Freundlich model were only recorded as 0.90–0.92 (Figure S6b). Accordingly, the Langmuir isotherm was more suitable to describe the current experimental data, revealing the mainly monolayer adsorption behavior for the MO capture [37]. As seen in Figure 6c, with the environment temperature increased from 25 to 45 °C, the corresponding adsorption capacities decreased (e.g., at an initial MO concentration of 100 mg L^−1^, the *Q*_e_ value decreased from 144.60 mg g^−1^ to 121.83 mg g^−1^) [10].

The effect of temperature on the adsorption activity was further investigated via the thermodynamics analysis. Several representative thermodynamic parameters such as the Gibbs free energy change (Δ*G*, kJ mol^−1^), enthalpy change (Δ*H*, kJ mol^−1^), and entropy change (Δ*S*, J mol^−1^ K^−1^) were determined by Equations 5, 6 and 7 [23,28].

(5) ΔG=−R·T·lnK

(6) $$K=\frac{\alpha_{\mathrm{ad}}}{\alpha_{e}}=\frac{\gamma_{\mathrm{ad}}\cdot C_{\mathrm{ad}}}{\gamma_{e}\cdot C_{e}}$$

(7) $$\ln K=\frac{\Delta S}{R}-\frac{\Delta H}{R\cdot T}$$

where *R* is the gas constant (8.314 J mol^−1^ K^−1^), *T* (K) is absolute temperature; *K* is the thermodynamic equilibrium constant, *γ*_ad_ and *γ*_e_ are activity coefficients, and *C*_ad_ is the MO concentration adsorbed on the sample (mg L^−1^). In the dilute solution, the activity coefficient approached unity, and the *K* value could be determined graphically by plotting ln(*C*_ad_/*C*_e_) against *C*_ad_ and extrapolating to zero [19].

Figure S7 displays the linear fitting result of relationship *C*_ad_-ln(*C*_ad_/*C*_e_). The *K* constants were calculated from the intercept with the Y-axis (4.275 at 25 °C, 2.449 at 35 °C, 1.642 at 45 °C). The Δ*G* values of 25, 35 and 45 °C could be obtained as −3.60, −2.29 and −1.31 kJ mol^−1^, respectively (Table S5). Negative Δ*G* values revealed the spontaneous nature of the adsorption reaction [38]. Furthermore, the Δ*G* values were usually in the range of 0 to −20 kJ mol^−1^ and −80 to −400 kJ mol^−1^ for physical and chemical adsorptions, respectively [39]. In this case, the MO adsorption onto the DCS/Fe_3_O_4_-Cu adsorbent was the physical adsorption (electrostatic force between Cu cations and MO anions) [39].

From Figure 6d, the Δ*H* and Δ*S* values were obtained from the slope and intercept of 1/*T*-ln*K* plots. Using Equation 7, the Δ*H* and Δ*S* values were calculated as −37.78 kJ mol^−1^ and −114.82 J mol^−1^·K^−1^. The negative Δ*H* value suggested the exothermic nature for this adsorption behavior, and low temperature was favorable for the adsorption reaction [38,39], which was consistent with the adsorption isotherms in Figure 5c.

### 2.3. Regeneration Study of DCS/Fe_3_O_4_-Cu

**Figure 7:**
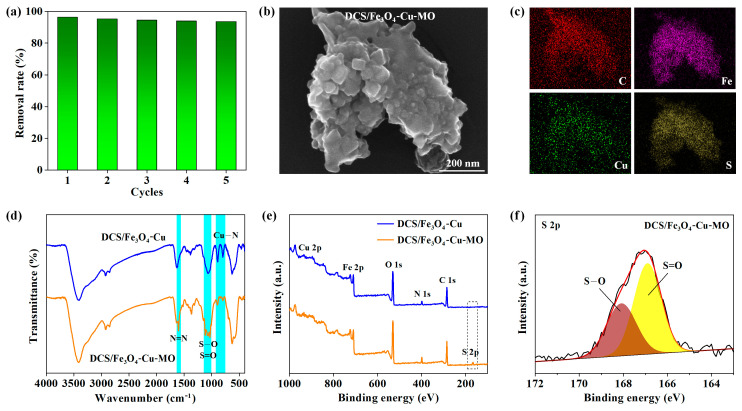
Properties of DCS/Fe_3_O_4_-Cu-MO (DCS/Fe_3_O_4_-Cu after 5 adsorption MO cyclings): (**a**) removal efficiency, (**b**) SEM image of DCS/Fe_3_O_4_-Cu-MO, (**c**) mapping images, (**d**) FTIR spectra of DCS/Fe_3_O_4_-Cu and DCS/Fe_3_O_4_-Cu-MO, (**e**) XPS survey spectra of DCS/Fe_3_O_4_-Cu and DCS/Fe_3_O_4_-Cu-MO, (**f**) high-resolution XPS spectrum of S 2p.

The good regeneration ability of the adsorbent is very important for the cost factor in industrial application [40]. Figure 7a expressed the removal rate of MO onto the DCS/Fe_3_O_4_-Cu sample during repeated absorption–desorption cycles (MO initial concentration of 40 mg L^−1^, environment temperature of 25 °C). For the first absorption process, the DCS/Fe_3_O_4_-Cu adsorbent delivered a high removal efficiency of 96.4%. Owing to the convenient recyclability from the magnetic Fe_3_O_4_, the removal rate was still recorded as 93.5% after five regeneration cycles.

The morphology of the DCS/Fe_3_O_4_-Cu material after five absorption cycles (labeled as DCS/Fe_3_O_4_-Cu-MO) is displayed in Figure 7b. The DCS/Fe_3_O_4_-Cu-MO sample still maintained the original agglomerated structure after multiple times absorption–desorption processes [9,25]. From Figure 7c, the mapping images exhibited uniform distribution of C, Fe and Cu elements of the DCS/Fe_3_O_4_-Cu-MO particle. The S element mapping image further confirmed the capture of MO molecules.

Figure 7d presents the FTIR spectra of DCS/Fe_3_O_4_-Cu and DCS/Fe_3_O_4_-Cu-MO samples. It could be found that both samples kept original characteristic peaks of the O–H bond (3480 cm^−1^), C–H bond (2900 cm^−1^), –NH_2_ bond (1640 cm^−1^), C–O bond (1080 cm^−1^), Fe–O bond (600 cm^−1^), and Cu–N bond (~890 cm^−1^). These characteristic peaks suggested the structural integrity of material during the absorption process [9]. After MO absorption, a new sharp peak located at 1600 cm^−1^ could be corresponded to the N=N bond in an adsorbed MO molecule [23,28]. Two new sharp peaks at 1030 and 1116 cm^−1^ were assigned to the S–O and S=O bonds in the MO molecule, respectively. These results also verified the successful absorption of MO onto the DCS/Fe_3_O_4_-Cu adsorbent.

The XPS survey spectra of DCS/Fe_3_O_4_-Cu and DCS/Fe_3_O_4_-Cu-MO samples are displayed in Figure 7e. The characteristic peaks of C 1s (285 eV), N 1s (399 eV), O 1s (531 eV), Fe 2p (710 eV), and Cu 2p (934 eV) were all retained after MO absorption. A new peak that appeared at ~167 eV on the DCS/Fe_3_O_4_-Cu-MO curve was the characteristic peak of S 2p [41]. The S 2p high-resolution XPS spectrum (Figure 7f) further displayed two peaks centered at 168.2 and 166.9 eV. The former peak could be attributed to the S–O bond, and the latter peak was indexed to be the S=O bond, respectively [42].

## 3. Materials and Methods

### 3.1. Materials and Reagents

Chitosan (CS, deacetylation degree of 96%), diethylenetriamine, epichlorohydrin (C_3_H_5_ClO), ethylenediamine tetraacetic acid (EDTA), FeCl_3_·6H_2_O, FeSO_4_·7H_2_O, CuSO_4_·5H_2_O, and methyl orange (MO) were purchased from Energy Chemical Industrial Inc. (Shanghai, China). Ammonia (NH_3_·H_2_O), acetic acid (CH_3_COOH) and ethanol (C_2_H_5_OH) were purchased from Sinopharm Chemical Reagent Co., Ltd. (Shanghai, China). All chemical reagents were analytical grade and used directly without further purification.

### 3.2. Synthesis of CS/Fe_3_O_4_

The CS/Fe_3_O_4_ was prepared by a one-step coprecipitation method, as shown in Figure 1a. Typically, 1 g of CS was dissolved in 200 mL of acetic acid solution with ultrasonic oscillation. Then, 4.7 g of FeCl_3_·6H_2_O and 2.4 g of FeSO_4_·7H_2_O were dissolved in 22 mL deionized water and added into the CS solution. After, 40 mL of NH_3_·H_2_O (28%) was dropwise added into solution under continuous stirring at 40 °C for 20 min. Subsequently, 6 mL of C_3_H_5_ClO was added into the solution at 60 °C for 3 h to form the CS/Fe_3_O_4_.

### 3.3. Synthesis of DCS/Fe_3_O_4_-Cu

As shown in Figure 1b, 1 g of CS/Fe_3_O_4_ and 3.1 g of diethylenetriamine were dissolved in 70 mL acetic acid solution plus 100 mL of deionized water under vigorous stirring at 80 °C for 12 h. The as-prepared DCS/Fe_3_O_4_ was added into CuSO_4_·5H_2_O solution to adsorb Cu ions (II). After washing with CH_3_COOH/C_2_H_5_OH/deionized water and drying, the magnetic DCS/Fe_3_O_4_-Cu product could be obtained.

### 3.4. Characterization

The crystalline structures of materials were measured by the AXS D8 Advance X-ray diffractometer (XRD, Cu Kα source; Bruker, Karlsruhe, Germany). The element compositions of samples were identified via the X-ray photoelectron spectrometry (XPS) using an ESCA LAB MK-II X-ray photoelectron spectrometer (VG Scientific, St Leonards, UK). The surface functional groups of materials were confirmed by Fourier transform infrared (FTIR) spectroscopy via the KBr pellet using a Nicolet iS10 spectrometer (Thermal Fisher Scientific, Waltham, MA, USA). The surface morphologies of samples were investigated by the field-emission scanning electron microscopy (FESEM, SU8010, Hitachi, Tokyo, Japan). The thermal stabilities of materials were tested on the Mettler–Toledo (Zurich, Switzerland) thermogravimetric (TG) Stare ESI-0910 instrument from room temperature (25 °C) to 900 °C (heating rate of 3 °C min^−1^) under Ar atmosphere. The specific surface areas and pore size distributions of samples were determined via N_2_ adsorption–desorption experiments using the Brunauer–Emmett–Teller (BET) method by an Autosotrb-IQ2-MP-XR (Micromeritics, Norcross, GA, USA) gas sorption analyzer (samples were degassed at 200 °C in vacuum for 18 h). The magnetic properties of materials were measured by a vibrating-sample magnetometer (VSM, PPMS DynaCool 9, Quantum Design, San Diego, CA, USA) at room temperature, and the hysteresis loops were recorded in the field range of 30,000 Oe. The zeta potential of samples was measured by a Zetasizer Nano Z with a surface zeta potential accessory (Malvern, Worcs, UK).

### 3.5. Adsorption Experiments

To prepare the DCS/Fe_3_O_4_-Cu, a certain amount of the DCS/Fe_3_O_4_ was added into 30 mL of CuSO_4_·5H_2_O solution. The solution pH (2–6, adjusted using 0.1 M H_2_SO_4_ or 0.1 M NaOH), adsorbent dosage (1–8 g L^−1^), initial Cu ions concentration (50–300 mg L^−1^), and contact time (0–700 min) were investigated. Using a thermostatic shaker (SHZ-C, Suzhou, China) at 25 °C/150 rpm during the whole adsorption process, the concentration of Cu ions was determined by the inductively coupled plasma optical emission spectrometer (ICP-OES, Avio 500, PerkinElmer, Waltham, MA, USA).

Similarly, for the batch adsorption experiments of MO, 0.015 g of CS/Fe_3_O_4_, DCS/Fe_3_O_4_ or DCS/Fe_3_O_4_-Cu adsorbent was added into 30 mL of MO aqueous solution (MO initial concentration of 10–100 mg L^−1^) at 25 °C. The MO concentration in solution was determined by an UV-3600 PLUS spectrophotometer (Shimadzu, Kyoto, Japan) at a wavelength of 460 nm. The removal efficiency (*R*, %) and adsorption capacity (*Q*_e_, mg g^−1^) can be calculated by the following Equation 8 and Equation 9, respectively.

(8) $$R=\frac{C_{0}-C_{e}}{C_{0}}\times 100\%$$

(9) $$Q_{e}=\frac{\left(C_{0}-C_{e}\right)\cdot V}{m}$$

where *C*_0_ (mg L^−1^) is the initial concentration of Cu^2+^ or MO, *C*_e_ (mg L^−1^) is the equilibrium concentration, *V* (L) is the volume of solution, and *m* (g) is the mass of adsorbent.

### 3.6. Desorption and Regeneration

The EDTA solution, C_2_H_5_OH, and ultrapure H_2_O were used as the desorption agents. For each adsorption/desorption cycle, the adsorbent (DCS/Fe_3_O_4_-Cu) after MO adsorption was separated and collected by a magnet. Adding a small amount of NaOH under ultrasonic oscillation for 20 min, the spent absorbent could be regenerated by thoroughly washing using the EDTA, C_2_H_5_OH plus ultrapure H_2_O several times and drying. Such a regenerated DCS/Fe_3_O_4_-Cu adsorbent was further used for the removal of MO in solution.

## 4. Conclusions

In this study, the CS/Fe_3_O_4_ material was prepared via Fe_3_O_4_ nanoparticles immobilized onto the CS matrix. After diethylenetriamine modification and the adsorption of Cu^2+^, the CS/Fe_3_O_4_ further formed the DCS/Fe_3_O_4_-Cu. The composite material displayed the sub-micron size of an agglomerated structure with numerous Fe_3_O_4_ nanoparticles (particle size of 50–100 nm). Due to the electrostatic force between bridging Cu cations and MO anions, the DCS/Fe_3_O_4_-Cu adsorbent exhibited an enhanced adsorption performance. For example, the DCS/Fe_3_O_4_-Cu delivered an excellent removal efficiency of 96.4% for MO at 40 min, which was much higher than 38.7% of the pristine CS/Fe_3_O_4_. At 100 mg L^−1^ MO initial concentration and 25 °C, the DCS/Fe_3_O_4_-Cu exhibited the maximum adsorption capacity of 144.60 mg g^−1^. The experimental data were well explained by the pseudo-second-order model and Langmuir isotherm, indicating the dominant monolayer adsorption for MO. A thermodynamic study confirmed the exothermic process of MO adsorption. The magnetic adsorbent can be easily separated and recycled from solution, so the DCS/Fe_3_O_4_-Cu still maintained a large removal efficiency of 93.5% after five regeneration cycles. This work constructed a magnetic DCS/Fe_3_O_4_-Cu adsorbent with high adsorption capability and convenient recyclability, which provided the potential application for wastewater treatment.

## Data Availability

Not applicable.

## Supplementary Materials

The following supporting information can be downloaded at: https://www.mdpi.com/article/10.3390/molecules28052307/s1, Figure S1: Parameter effects of Cu^2+^ removal efficiency for CS/Fe_3_O_4_; Figure S2: BJH pore size distributions of samples; Figure S3: EDS spectra of samples; Figure S4: UV-Vis absorbance spectra of MO adsorption; Figure S5: Linear fitting of pseudo-first-order kinetic model; Figure S6: Linear fitting of Langmuir and Freundlich models; Figure S7: Thermodynamics plots of MO adsorption; Table S1: Grain sizes and crystallinity index parameters; Table S2: Pore texture parameters; Table S3: Kinetic parameters of MO adsorption; Table S4: Isotherm parameters of MO adsorption; Table S5: Thermodynamic parameters of MO adsorption.

## Sample Availability

Samples of the compounds are not available from the authors.
