# Supplementary material for: Recyclable Magnetic Iron Immobilized onto Chitosan with Bridging Cu Ion for the Enhanced Adsorption of Methyl Orange
Source: Molecules. 2023 Mar 2;28(5):2307. doi: 10.3390/molecules28052307 (PMC10005193; doi:10.3390/molecules28052307)
Supplement: Supplementary file 1 [file molecules-28-02307-s001.zip › molecules-2210012-supplementary.pdf]

Supporting Information

Recyclable Magnetic Iron Immobilized onto Chitosan with Bridging Cu Ion for the Enhanced Adsorption of Methyl Orange

Daoguang Teng [1,2], Peng Jin [2], Wenhuan Guo [2], Jiang Liu [1,2], Wei Wang [1,2], Peng Li [2], Yijun Cao [1,2], Ling Zhang [1,2,*] and Ying Zhang [1,2,*]

[1] Zhongyuan Critical Metals Laboratory, Zhengzhou University, Zhengzhou 450001, China

[2] School of Chemical Engineering, Zhengzhou University, Zhengzhou 450001, China

* Correspondence: zhang_ling@zzu.edu.cn (L.Z.); zhangying777@zzu.edu.cn (Y.Z.)

Supplemental Information is available free, including 7 figures and 5 tables.

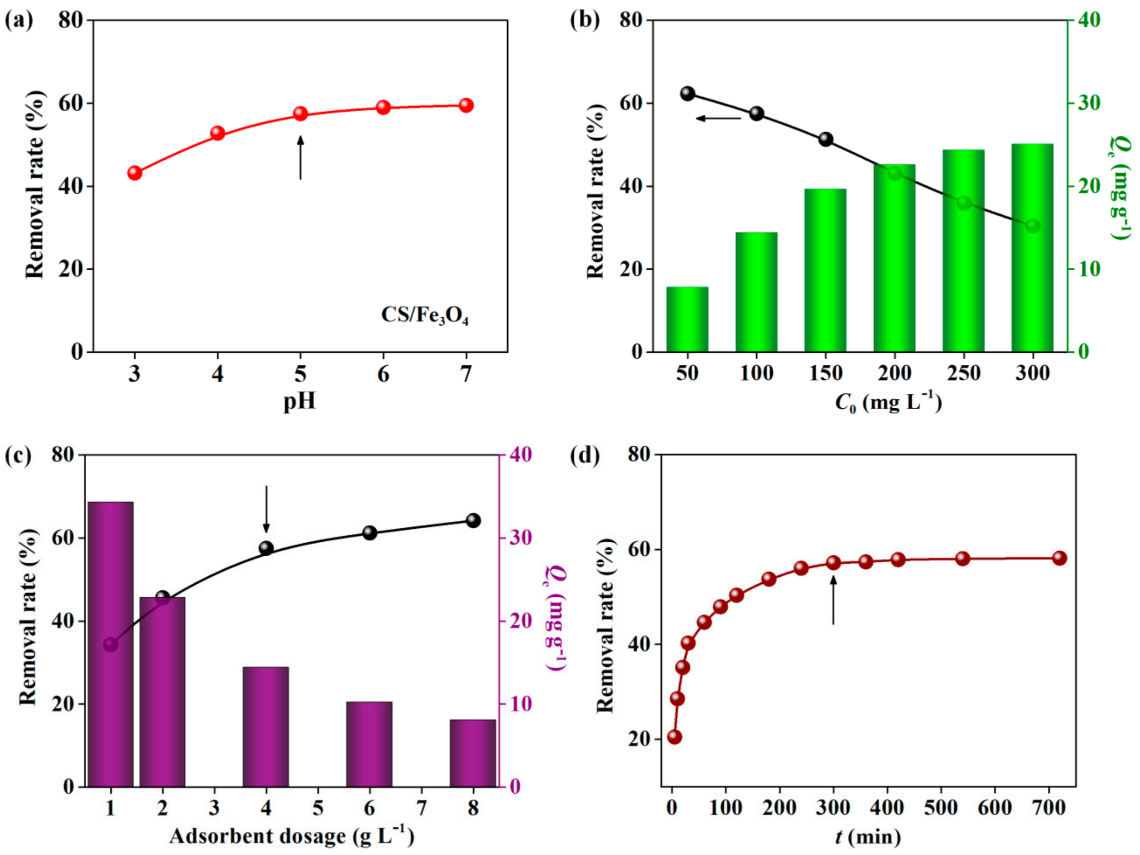

Figure S1. Effect of (a) pH value, (b) initial Cu^{2+} concentration, (c) absorbent dosage, and (d) contact time on Cu^{2+} removal efficiency for CS/Fe_3O_4.

From **Figure S1a**, the Cu^{2+} removal efficiency increased with the reduction of pH value. At pH = 5, the removal efficiency reached the saturated value (~60%). **Figure S1b** showed that Cu^{2+} removal rate decreased with the increasement of initial Cu^{2+} concentrations (50–300 mg L^{-1}), while the corresponding adsorption capacity increased. As seen in **Figure S1c**, with the absorbent dosage increased from 1 to 8 g L^{-1}, the Cu^{2+} removal rate increased from 34.3% to 64.2%. With the prolonging of contact time, the Cu^{2+} removal efficiency gradually increased (**Figure S1d**). Hence, the optimal conditions for CS/Fe_3O_4 to remove Cu^{2+} were pH = 5, Cu^{2+} concentration = 100 mg L^{-1}, absorbent dosage = 4 g L^{-1}, and contact time = 300 min.

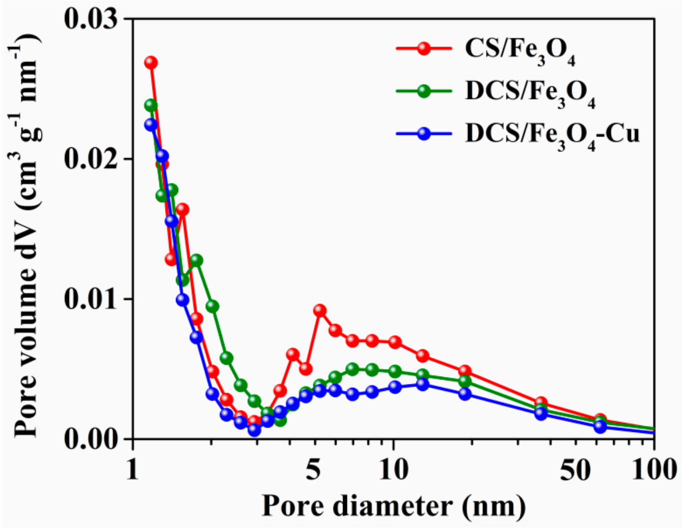

Figure S2. The Barrett-Joyner-Halenda (BJH) pore size distributions of CS/Fe_3O_4, DCS/Fe_3O_4, and DCS/Fe_3O_4-Cu.

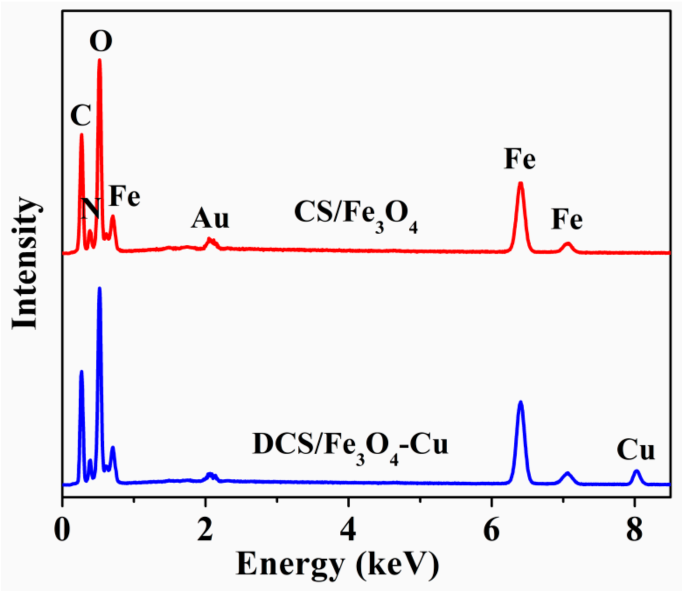

Figure S3. The EDS spectra of CS/Fe_3O_4 and DCS/Fe_3O_4-Cu.

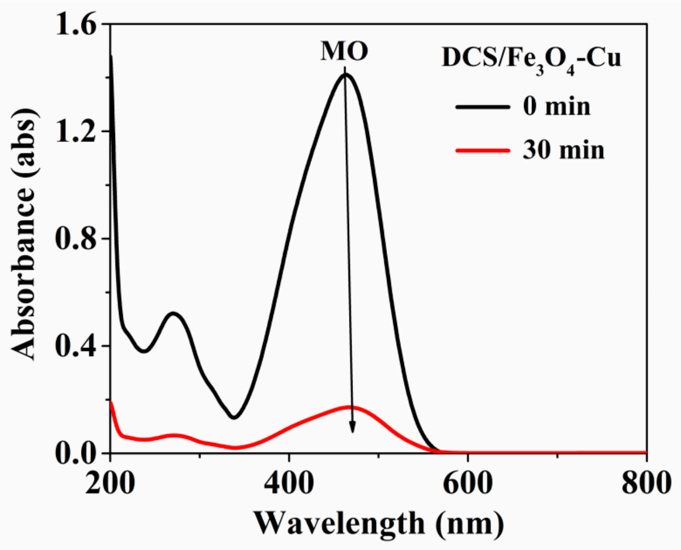

Figure S4. The UV-Vis absorbance spectra of MO adsorption onto DCS/Fe_3O_4-Cu.

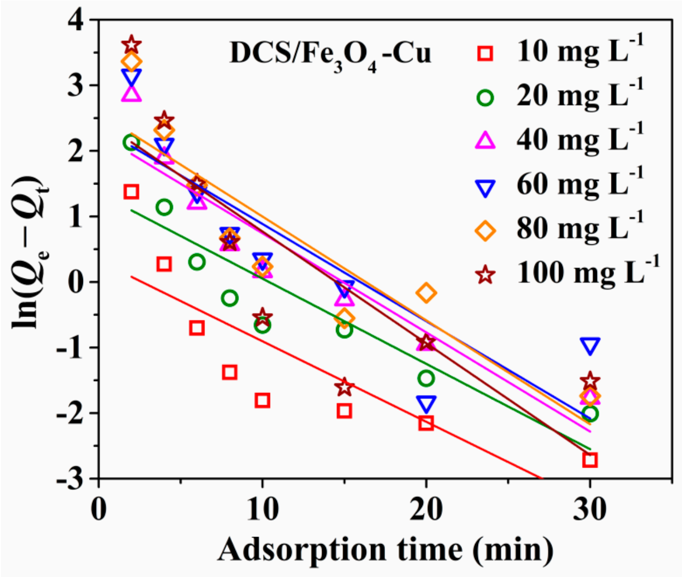

Figure S5. Linear fitting of pseudo first-order kinetic model for DCS/Fe_3O_4-Cu.

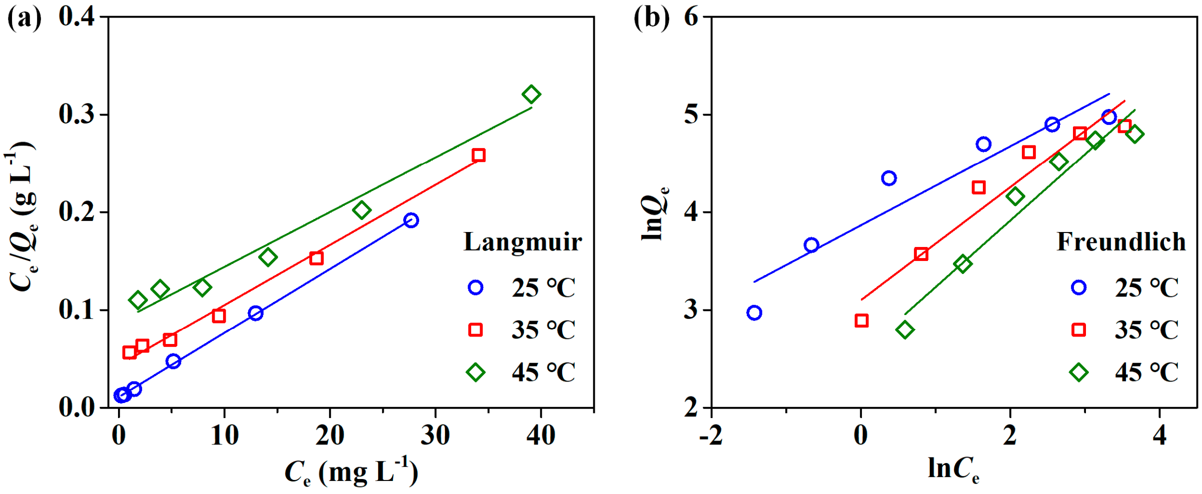

Figure S6. Linear fitting of (a) Langmuir and (b) Freundlich models for DCS/Fe_3O_4-Cu.

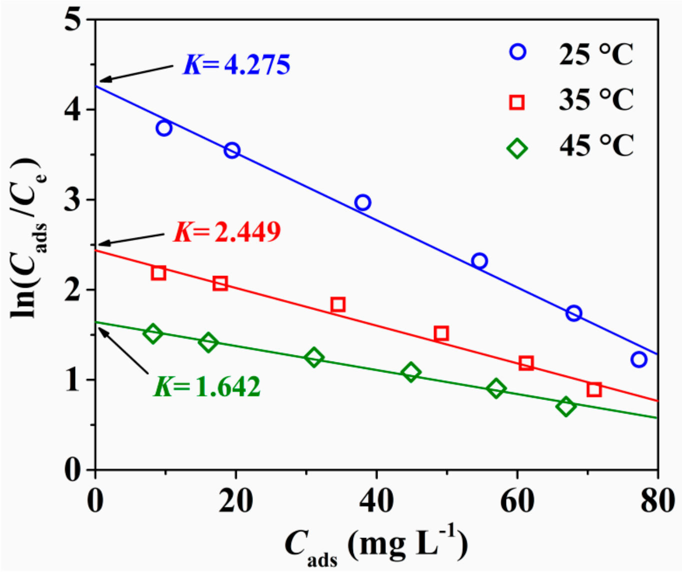

Figure S7. Thermodynamics plots of MO adsorption onto DCS/Fe_3O_4-Cu.

Table S1. Grain sizes and crystallinity index parameters of four samples.

Samples	Grain size (nm)	Crystallinity index
CS	3.4	63%
CS/Fe_3O_4	8.2	77%
DCS/Fe_3O_4	12.4	82%
DCS/Fe_3O_4-Cu	27.9	86%

Table S2. Pore texture parameters of three samples.

Samples	S_{BET} (m^2 g^{-1})	V_t (cm^3 g^{-1})	d_a (nm)
CS/Fe_3O_4	38.4	0.198	20.6
DCS/Fe_3O_4	28.7	0.154	21.5
DCS/Fe_3O_4-Cu	25.8	0.142	22.0

Remark: S_{BET} — the specific surface area using the BET equation;

V_t — the total pore volume at relative pressure P/P_0 of 0.99;

d_a — average pore diameter was calculated as: d_a=4000V_t/S_{BET}.

Table S3 Kinetic parameters of MO adsorption onto DCS/Fe_3O_4-Cu.

C_0	Q_e (25 °C)	Pseudo second-order		
(mg L^{-1})	(mg g^{-1})	Q_{e2} (mg g^{-1})	K_2 (g mg^{-1} min^{-1})	R^2
10	19.52	19.70	0.1718	0.998
20	38.97	39.37	0.0694	0.999
40	77.09	78.12	0.0315	0.999
60	109.66	111.11	0.0254	0.998
80	134.08	135.14	0.0211	0.998
100	144.60	147.06	0.0178	0.997

Table S4 Fitting isotherm parameters of MO adsorption onto DCS/Fe_3O_4-Cu.

T (°C)	Langmuir model			Freundlich model		
	Q_m (mg g^{-1})	K_L (L mg^{-1})	R^2	n	K_F (mg g^{-1})(L mg^{-1})$^{1/n}$	R^2
25	163.25	0.5046	0.996	2.467	47.72	0.905
35	152.60	0.1418	0.983	1.731	22.19	0.910
45	147.67	0.0638	0.971	1.471	12.87	0.921

Table S5 Thermodynamic parameters of MO adsorption onto DCS/Fe_3O_4-Cu.

T (K)	ΔG (kJ mol^{-1})	ΔH (kJ mol^{-1})	ΔS (J mol^{-1} K^{-1})
298.15	−3.60		
308.15	−2.29	−37.78	−114.82
318.15	−1.31		
